# Hiatal Hernias Revisited—A Systematic Review of Definitions, Classifications, and Applications

**DOI:** 10.3390/life14091145

**Published:** 2024-09-11

**Authors:** Karl Hermann Fuchs, Ioannis Kafetzis, Alexander Hann, Alexander Meining

**Affiliations:** 1 Laboratory for Interventional and Experimental Endoscopy (InExEn), University of Würzburg, Grombühlstr. 12, 97080 Würzburg, Germany; kafetzis_i@ukw.de (I.K.); hann_a@ukw.de (A.H.); meining_a@ukw.de (A.M.); 2 Head of Gastroenterology, Zentrum Innere Medizin, University of Würzburg, 97080 Würzburg, Germany

**Keywords:** hiatal hernia, para-oesophageal hernia, GERD, fundoplication, upside-down stomach

## Abstract

Introduction: A hiatal hernia (HH) can be defined as a condition in which elements from the abdominal cavity herniate through the oesophageal hiatus in the mediastinum and, in the majority of cases, parts of the proximal stomach. Today, the role of HHs within the complex entity of gastroesophageal reflux disease (GERD) is very important with regard to its pathophysiology, severity, and therapeutic and prognostic options. Despite this, the application and stringent use of the worldwide accepted classification (Skinner and Belsey: Types I–IV) are lacking. The aim of this study was to carry out a systematic review of the clinical applications of HH classifications and scientific documentation over time, considering their value in diagnosis and treatment. Methods: Following the PRISMA concept, all abstracts published on pubmed.gov until 12/2023 (hiatal hernia) were reviewed, and those with a focus and clear description of the application of the current HH classification in the full-text version were analysed to determine the level of classification and its use within the therapeutic context. Results: In total, 9342 abstracts were screened. In 9199 of the abstracts, the reports had a different focus than HH, or the HH classification was not used or was incompletely applied. After further investigation, 60 papers were used for a detailed analysis, which included more than 12,000 patient datapoints. Among the 8904 patients, 83% had a Type I HH; 4% had Type II; 11% had Type III; and 1% had Type IV. Further subgroup analyses were performed. Overall, the precise application of the HH classification has been insufficient, considering that only 1% of all papers and only 54% of those with a special focus on HH have documented its use. Conclusions: The application and documentation of a precise HH classification in clinical practice and scientific reports are decreasing, which should be rectified for the purpose of scientific comparability.

## 1. Introduction

The term “hiatal hernia” (HH) was established as a clinical entity when radiography proved its existence and enabled it to be applied it as a diagnosis [1,2,3]. HH can be defined as a condition in which elements from the abdominal cavity herniate through the oesophageal hiatus in the mediastinum and, in the majority of cases, parts of the proximal stomach [1,2,3]. The introduction of HH as a diagnosis based on clinical signs was challenging, as it was difficult to associate specific symptoms with radiographic findings [1,2,3]. Initially, the diagnosis of an HH as an underlying cause of problems was limited to rare events such as traumatic injuries, intrathoracic volvulus, and strangulations [1,2]. With the exception of such emergencies, the clinical presentation of patients with a variety of foregut symptoms, such as epigastric pain and burning, thoracic pain and burning, belching, regurgitation, heartburn, nausea, and vomiting, could not be directly associated with the presence of hiatal hernias [1,2,3]. These symptoms had at the time a substantial overlap with other disorders of the foregut, such as gastro-duodenal ulcer disease [2,3,4]. As a consequence, the attention of physicians to the presence of an HH was limited until an association emerged in patients with reflux-like symptoms such as heartburn, regurgitation, and epigastric pain [2,3,4,5,6].

In 1926, Akerlund introduced an initial classification of the different anatomical elements and changes in the hiatal anatomy [1,2]. He documented three main elements of hiatal hernia classification: the first group of hernia patients frequently showed a shortened oesophagus, with the stomach partially in the chest above the level of the diaphragm. At the time, this condition was subject to widespread discussion because some authors suspected a congenital short oesophagus. In the second type of hernia, a normal length of the oesophagus and a para-oesophageal herniation of part of the stomach through a small defect in the phreno-oesophageal attachments were observed, rolling up next to the oesophagus in the mediastinum. In the third group of patients, the proximal stomach was observed to be gradually sliding up and herniating into the mediastinum. In this latter group, the oesophago–gastric junction did not remain at the hiatus because of a circumferential weakening of the attachments at the hiatus, lifting the cardia above the level of the diaphragm. These three groups describing and defining the different anatomical changes remain valid today [1,2]. 

The 1940s saw more detailed work on and increasing attention paid to patients with hiatal hernia, especially by Allison, Sweet, and Barrett [7,8,9,10,11,12,13]. These reports stimulated the clinical and research interests of internists and surgeons in the late 1940s and 1950s [7,8,11,12,13]. Allison analysed different anatomical types of HH in another attempt to classify the changes [7]. He recognised and subsequently popularised the association between the presence of HHs and reflux symptoms such as heartburn and regurgitation, as well as the presence of reflux esophagitis and its surgical therapy [7,8,9,10,11,12]. Barrett focused on the functional aspect of the cardia and the functionality of the “angle of His” at the distal oesophagus as a critical element of reflux prevention [13]. 

Akerlund’s and Allison’s classifications were the basis for definitions and documentation throughout the 1950s and 1960s and were used to structure the data of clinical studies until Skinner and Belsey published their milestone work on over 1000 patients with HHs and reflux in 1967, creating, based on their experience, the basis for the most frequently used HH classification in the past 60 years [7,8,9,10,11,12,13,14,15,16,17,18,19,20,21,22,23]. Skinner used the elements of HH typing from Akerlund’s analysis and added two more elements, summarised in the Type IV hernia: the description of an upside-down stomach and the migration of other organs such as the colon, spleen, and/or pancreas into a large intrathoracic hernia sac [23]. This classification was accepted worldwide and was applied with sufficient precision over the first two or three decades until the 1990s. The different types of HH reflect important information, as there is a direct association between some HH types and GERD in terms of the pathophysiology and severity of the disease, as well as therapeutic decision-making regarding the choice of medical or operative therapy and the choice of surgical procedure.

Unfortunately, today, there is not only an increasing lack of discipline when classifying hiatal hernias according to the well-accepted classification proposed by Skinner and Belsey but also a change in the taxonomy in many publications that report undefined terms, such as “massive”, “giant”, or “large” hernias.

The aim of this study was to carry out a systematic review of the clinical applications of HH classifications and scientific documentation over time, considering their value for diagnosis and treatment.

## 2. Methods

The design of our study and review was based on the search for available classifications throughout the documented history of HH reports. It was important to identify the major elements of classifications in the various anatomical HH types and their use in clinical practice, their ongoing use as valuable tool for differentiation, and their disappearance in subsequent publications because of a lack of clinical importance. The intention was to investigate the correct application of HH classifications, as well as their distribution, in order to determine the incidence of HH typing in published series. In addition, our aim was to document deviations from the established classifications and determine the possible effect on the usability of the published results. 

Therefore, we performed a literature search using the term “hiatal hernia” in pubmed.gov in 2023. We followed the PRISMA concept for this project. All available abstracts were investigated from 1939 to 2023. A special effort was made to expand the search in order to obtain abstracts and text publications that were crucial in terms of their historical role and relevant results related to the correct application of classifications, their stringency, and the distribution of different anatomical types of hiatal hernias among the published cohorts.

All abstracts were read and searched for relevant information on the application of any hiatal hernia classification, different typing of HHs, the distribution of established HH types for a comparison with other reports, and documentation of unusual HH taxonomy. Special attention was paid to the first-time use of any new definitions and/or classifications, the results of their application, and the possible advantages and reasons for the application of alternative typing methods.

The use of different established classifications in each decade starting from the 1930s to 2023 was also documented. In addition, all different elements characterising a hiatal hernia were registered, and their applications in the different publications were documented. These elements are presented in Table 1.

**Figure 1:**
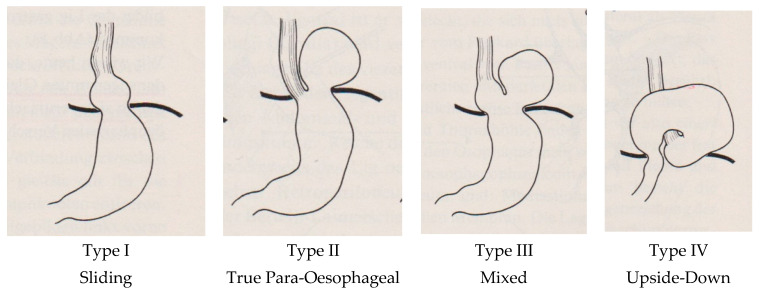
Established typing of hiatal hernias by Skinner and Belsey in 1967, subdivided into four types [23].

These elements were searched in every publication for their presence and documentation, as well as their frequency of appearance. The basis of classification was the initial version of Akerlund, expanded by Allison and finalised by Skinner and Belsey [1,2,3,7,11,12,13,23], as demonstrated in Figure 1.

Finally, these data were analysed in relation to the number of patients included in the different series. Some early publications from the 1930s and 1940s provided only a descriptive overview using the correct classification elements but no detailed data, and these were excluded from the data analysis.

## 3. Results

In total, 9342 abstracts were screened. The vast majority of the papers had HH as one of the keywords and therefore appeared in the abstract list but had a different focus, with no relevant information or data regarding HHs. Another substantial proportion of the publications, especially among those from the most recent two decades, had an associated focus such as gastroesophageal reflux disease (GERD), but lacked any information about the presence, prevalence, or incidence of HHs in the investigated population of patients or persons.

**Figure 2:**
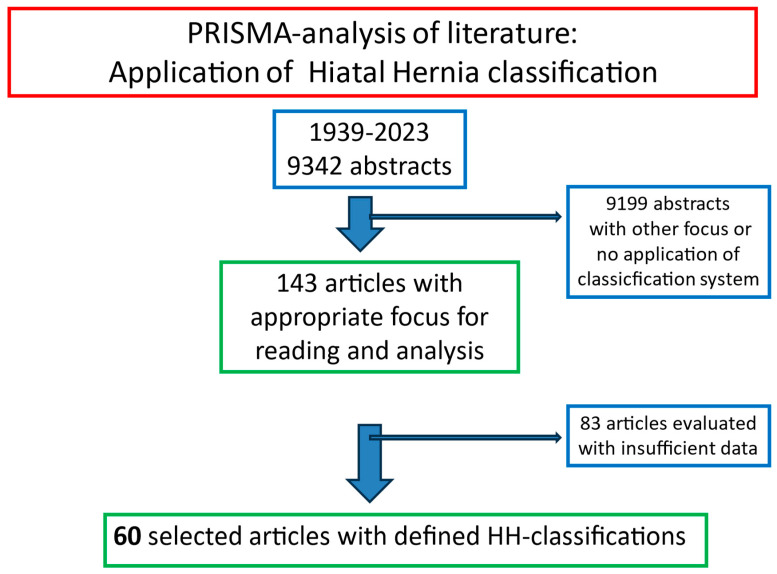
PRISMA selection of 60 abstracts and original reports on hiatal hernias published between 1926 and 2023 in pubmed.gov.

Out of the 9342 abstracts, 9199 abstracts had either a different focus than HH or an HH classification was not used (Figure 2). Thus, these abstracts were excluded from further calculations. For the remaining 143 abstracts, an in-depth reading of the original published manuscript was performed. The documented distributions of the different HH types were extracted and registered. After a detailed analysis seeking the correct application of the HH taxonomy and documented typing, 60 manuscripts were selected for the final evaluation [10,13,15,17,18,20,21,22,23,24,25,26,27,28,29,30,31,32,33,34,35,36,37,38,39,40,41,42,43,44,45,46,47,48,49,50,51,52,53,54,55,56,57,58,59,60,61,62,63,64,65,66,67,68,69,70,71,72,73,74,75].

The analysis of the publications revealed the initial effort made between the 1920s and the 1960s to detect and create an understanding of HHs and their diagnostic challenges, as well as the difficultly in establishing an association with presenting clinical symptoms. In the 1960s, with the increasing understanding of the pathophysiology of reflux and the associated pathologic changes, the research moved away from the anatomy of HHs and towards the detection and interpretation of functional alterations in the oesophagus and stomach.

Researchers between 1930 and 1960 struggled with the interpretation of different anatomical diaphragmatic hernia variations [1,2,3,4,5,6,7,8,9,10,11,12,13,14,15,16]. As there could be substantial discrepancies between the size of a hiatal hernia and the associated symptoms, and considering the limited diagnostic armamentarium, patients with post-traumatic hernias often presented seeking medical help [1,2,3]. At the time, some alterations in anatomy were still believed to be congenital defects rather than acquired conditions based on pathologic developments [2,3,4,5,6,7,8,9,10,11,12,13]. 

The clinical findings of an intraluminal visible shortened squamous epithelium appearing as a “brachyoesophagus” in an oesophagus with a normal length, or a true shortened oesophagus where the cardia did not reach below the diaphragm, were initially confusing and difficult to interpret [1,2,3,7,8,9,10,11,12,13]. Allison and Barrett clarified these findings in their landmark publications [7,13]. As a result, very few systematic applications of HH classifications were documented until the 1950s, and Kaplan et al. were the first to classify their patients accurately [11]. With the acceptance of the Skinner and Belsey classification of four types of hiatal hernias, more applications were systematically performed in the 1960s [23,24,25,26,27,28,29,30,31,32,33].

As a result of the analysis of 60 publications, a complete typing of all patients within a published cohort, usually of GERD and HH patients, was systematically and frequently performed between the 1960s and the late 1990s [23,24,25,26,27,28,29,30]. In addition, another trend could be observed in the focus of publications from the 1990s until today, which especially focused on the so-called “para-oesophageal” hernias [30,31,32,33,34,35,36,37,38,39,40,41,42,43,44,45,46,47,48,49,50,51,52,53,54,55,56,57,58,59,60,61,62,63,64,65,66,67,68,69,70,71,72,73,74,75,76,77]. The analysis showed that, in recent decades, the majority of publications have focused on the surgical therapy of para-oesophageal hernias (PEHs), unfortunately quite often without further differentiation between a true PEH (Type II), a mixed (Type III), or a Type IV hernia. 

Out of the initially 77 selected original manuscripts, 17 publications focused only on the description of all different types but did not provide detailed data. These were usually early publications, when the authors struggled for definitions, describing the clinical experience from 1739 patients [1,2,3,4,5,6,7,8,9,11,12,14,16,19,20,76,77]. These were also excluded from the detailed data analysis.

The remaining 60 selected publications could be separated into two groups: a group with differentiation of all HH types and a second group with a focus on selective PEHs [10,13,15,17,18,20,21,22,23,24,25,26,27,28,29,30,31,32,33,34,35,36,37,38,39,40,41,42,43,44,45,46,47,48,49,50,51,52,53,54,55,56,57,58,59,60,61,62,63,64,65,66,67,68,69,70,71,72,73,74,75]. Table 2 shows the distribution of the publications and the corresponding number of patients examined.

Some publications used the term PEH for just Type II [35,39], some only included patients with Type IV hernias [37], some included Types III and IV [44,45], and the majority considered Types II, III, and IV. In addition, for the terms massive or giant PEH, a variety of further sizing was applied, such as HHs with vertical measurements of >3 cm or >5 cm or percentages of stomach above the diaphragm of >30%, >50%, or >75%.

The detailed results of the distribution of HH typing among the patient cohorts are given in Table 3, and they are given separately for the publications in which a complete cohort of all patients with HH was investigated, documented, and published, including 8904 patients. The latter showed a distribution between the sliding hernia of 7441 patients, a smaller group of 413 patients with Type II, 965 patients with a mixed type, and 85 patients with a Type IV HH. In addition, a further 3829 patients were analysed within cohorts with the exclusion of Type I patients, focusing only on selective PEH patients. The diagnosis of HH was established mainly using radiology until the 1960s and then gradually using upper GI endoscopy. 

These results confirmed that the vast majority of HH patients had a sliding hernia along the axial axis through the hiatal opening in the diaphragm, often participating in the pathophysiology of GERD. The distribution of the HH Types II, III, and IV varied, and the mixed type (mixed between para-oesophageal and sliding orally) was the most frequent HH among the PEH group. Both Type II, a true para-oesophageal hernia with the oesophago–gastric junction still at the hiatus, and Type IV, either an upside-down stomach or the migration of other organs into the chest, such as the pancreas, spleen, and colon, were rare findings, which may have needed different surgical approaches. 

The focusing of the distribution of these types in the two different time segments of publications, either the documentation of all HHs or the selection of only PEHs, showed some small differences. While the distribution in the selective PEH cohort between Types II, III, and IV was around 9%, 77%, and 13%, respectively, a similar calculation for the distribution of patients within the complete HH cohort for Types II, III, and IV was 28%, 66%, and 6%, respectively. 

**Figure 3:**
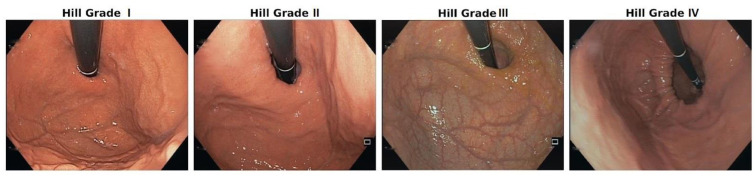
Endoscopy-based Hill classification: Grade I: normal situation; the mucosa fold is tight around the scope at the EGJ. Grade II: the mucosa fold is less prominent, showing a small space between the scope and mucosa, indicating a weak EGJ. Grade III: there is substantial space between the weakening mucosa and the scope, showing a small hiatal hernia and a widened hiatus. Grade IV: migration of the EGJ into the mediastinum, with a substantial hiatal hernia through the widened hiatus.

The Hill classification is an HH classification that can be established during upper GI endoscopy via visualisation of the cardia in retroflexion and the grading of anatomical changes (Figure 3) [41]. It is an excellent parameter for the initial widening and weakening of the EGJ (Grades I and III); however, other and larger anatomical alterations cannot be differentiated and are summarised as Grade IV [23].

This review shows that differentiation using all types has been neglected in the past two decades in most publications, despite the potentially varying pathophysiologic background and requirement for different therapeutic responses. Furthermore, the results demonstrate that a variety of definitions regarding size are in clinical use, which may interfere with the correct classification of cohorts. In 1983, the term “massive hernia” was first used in the published literature, without further explanation, for 47 patients with mixed (Type III) and 47 with Type IV hernias [35]. Since then, terms such as “large”, “massive”, and “giant” HH have often been used to describe the size of an HH, without clear criteria. These studies, unfortunately, cannot be used for scientific evaluations and comparisons, as precise definitions are necessary [44,46,47]. Overall, the precise application of HH classification has been insufficient, considering that only 1% of all papers and only 54% of those with a special focus on HH documented these details.

## 4. Discussion

Stylopoulos and Rattner summarised the early insights into hiatal hernias and the history of surgical therapy [78]. The authors described the increasing understanding of this radiologic finding and the increasing insights into the disease over the decades [78]. The development and presence of a hiatal hernia (HH) is one of the three most important factors in the pathophysiology of gastroesophageal reflux disease (GERD) [79,80,81,82]. Furthermore, an increase in size may contribute to the progression of GERD alongside the malfunctioning of the oesophago–gastric junction (EGJ) and pathologic intra-oesophageal acid exposure [82]. In addition, in some of these patients, the mechanical effect caused by the gastric “plug” in the mediastinum irritating the heart may be responsible for arrythmias or atrial fibrillation, which may require anatomical correction via surgery. As a consequence, the precise description of a cohort of patients should include information about the different types of hernias in the cohort and their size. As the established types of Skinner and Belsey have served well as a precise anatomical differentiation, physicians should utilise these terms. This information is valuable for the following reasons: First, it may allow for an assessment of the severity of the disease and thus provide the GERD patient with a prognosis. Second, the precise type and size of an HH may be important in determining the level of surgical experience and team necessary to perform the operation with a high probability of a good outcome. Third, the precise assessment and documentation of patient data may also be valuable not only for further scientific evaluation and learning through comparisons with data from other cohorts but also for quality control within the basic cohort. This should be a prerequisite at any academic institution.

The results of this literature review show that these recommendations are not followed stringently, and the majority of patient reports did not utilise an accepted HH classification. The most frequently used method of detection was upper GI endoscopy after the 1960s, which, until 2000, was often combined with radiography.

One reason for the failure to more accurately describe the type of HH could be the changing focus of research over the decades. Until the 1960s, surgeons sought a reasonable and practice-oriented classification of the different HH types, and Skinner and Belsey seemed to have found the solution [1,2,3,4,5,6,7,8,9,10,11,12,13,14,15,16,17,18,19,20,21,22,23]. In the next two decades, the focus of research turned to better understanding the functional and pathophysiologic components of GERD, supported by the increased possibility for functional assessment due to technological advances [83,84,85]. In the 1990s, surgical research focused on the technical introduction of the emerging minimally invasive procedures and their establishment in clinical practice [86,87,88]. More technical innovations and industrial marketing for the introduction of special devices and assessment tools followed in the 2000s, steering surgeons’ interest further away from HHs and their variations [89,90,91]. 

Within the increase in publications in the last 20 years on surgical therapy for PEHs, there seems to have been competition to publish the largest series on PEHs in the literature, despite the fact that this HH type is rather rare [23,24,25,26,27,28,29,30,31,32,33,34,35,36,37,38,39,40,41,42,43,44,45,46,47,48,49,50,51,52,53,54,55,56,57,58,59,60,61,62,63,64,65,66,67,68,69,70,71,72,73,74,75,76,77]. There are controversial applications of the term “PEH” and no precise definitions of size measurements, which prevents reasonable comparison among these studies.

Another reason is undoubtedly the role of endoscopy and oesophagitis as the main focus of interest in patients with GERD and/or foregut symptoms [92]. Endoscopists have frequently used Skinner’s borderline definition of HH size > 3 cm to define large hernias and <3 cm to define small hernias [23]. However, more often, superficial descriptions and terminology, such as “minor” or “extensive” oesophagitis, were found, which also lack precision. It must be emphasised that a more precise evaluation of vertical measurements can be achieved when entering the oesophagus and taking measurement on the way towards the stomach, with minimal air insufflation, compared with measurement on the way back, when a substantial amount of air/gas has been insufflated, thus changing the size of the stomach.

Some surgeons believe the anatomical alterations at the hiatus to be a purely mechanical process, similar to other abdominal hernias, and disconnected from possible functional alterations within the EGJ causing GERD [89]. Their concept of repair is based on mechanical closure of the hiatal gap using a durable mesh, without considering any functional measure, such as an augmentation of the lower oesophageal sphincter by a fundoplication [93]. Contentious discussions based on controversial study results have characterised the meetings around this subject, with a long history since the early 1950s, when the two approaches for interpreting GERD problems in association with HHs originated [7,12,79,81,93]. Thus, these two concepts, on the one hand, anatomical reconstruction by reducing the HH and, on the other hand, the more functional based approach of shaping and augmenting EGJ structures for reflux prevention, remain controversial [81]. Some surgeons remain unclear regarding the most effective anti-reflux procedure, that is, whether to only reconstruct the anatomy or add a fundoplication to the weak cardia [93]. However, specialised oesophageal surgeons recognise the multifactorial pathophysiologic background of GERD and combine HH repair with a fundoplication to respond to both anatomical and functional defects [79,81,82].

It must be emphasised that pathological reflux is associated with an increasing deterioration of both components of the EGJ within the hiatus, diaphragm connection, and lower oesophageal sphincter (LES) [82]. The circumferential weakening of the phreno-oesophageal membrane or ligament sets the initial basis for a sliding hernia, usually caused by a predisposing weakness of the membranous structure, based on overeating, exaggerated fundic overstretching, and chronic effacement of the LES, leading to shortening and functional weakness and weakening of the phreno-oesophageal membrane, ultimately causing hiatal herniation [81,82]. The two HH types involved in this process are Type I (sliding) and Type III (mixed). The latter belongs to the heterogenic group of PEHs (Types II, III, and IV), which are classified often within PEHs, even though their pathophysiologic origin and therapeutic approach are quite different from those of other PEHs [23,81].

In contrast, Type II (true PEH) and Type IV hernias (upside-down stomach and herniation of other organs) have no association with GERD, because, by definition, in these two HH types, the EGJ remains at the hiatal level, as only a small weak area within the circumference of the phreno-oesophageal membrane allows for herniation of the fundus [7,23,81]. These patients do not have substantial reflux. Depending on the local weakness of the tissue structures, the complete stomach may rotate and roll up into the hernia sac in the chest, resulting in an upside-down stomach [23]. Again, the EGJ stays at the hiatal level [7,23,81]. If the mobility of the neighbouring structures and organs, such as the spleen, colon, and pancreas, occurs, these may also follow the greater curvature of the stomach into the chest. This condition requires sufficient surgical expertise to perform a durable repair [23,26,49,56,59,71,76]. Thus, this condition must be recognised and diagnosed (HH typing) before making a therapeutic decision for surgery and selecting the appropriate team for the procedure.

There are essential differences within the PEH group, and, if these patients are not differentiated, a reasonable evaluation and scientific interpretation are not possible. Some authors have recently tried to simplify the definitions of PEHs: “a PEH exists when the fundus of the stomach is in a higher intrathoracic position than the EGJ” [94]. Another recent consensus project resulted in the following published statement: “There was an agreement (91%) that Para-esophageal Hiatal Hernia should be defined as the presence of a hernia sac extending from the abdominal cavity and/or bursa omentalis through the hiatus into the para-esophageal mediastinum and containing a variable portion of stomach…” [95]. These definitions facilitate its differentiation because they leave a difficult item out; if the cardia is still at the hiatal level, it probably prevents reflux caused by the intact LES function and partial diaphragmatic support. However, the latter factors seem to be very important in differentiating Types II, III, and IV and their corresponding roles in GERD. To the authors, this seems more important than the size of the HH and/or the extent of the para-oesophageal portion of stomach in the chest, as the surgical principal concept of correction is similar regardless of whether 30%, 50%, or 75% of the stomach is above the diaphragm. The latter argument may be a matter of controversy among surgeons.

As mentioned above, most HHs are diagnosed during the initial endoscopy during the GERD diagnostic work-up. Usually, endoscopy reports do not mention whether a certain protocol was used to carry out the size measurements and typing of the HH with minor insufflation in order to ensure an objective assessment. If the assessment was completed at the end of the endoscopy, after full insufflation, the data may be altered and not objective. Thus, endoscopic assessment can be valuable, but a strict protocol is necessary. 

The Hill classification, as mentioned above, is an HH classification that is established during upper GI endoscopy through visualisation of the cardia in retroflexion and the grading of anatomical changes [41]. In contrast to other classifications, it allows for a precise assessment of the EGJ, especially its early widening and increasing failure in early GERD by describing in Grade II and Grade III the gradual weakening of the cardia. Therefore, it is an excellent addition to the Skinner and Belsey classification. Unfortunately, it does not differentiate the major elements of differentiation in the more advanced anatomic alterations, such as HH Types II, III, and IV. Despite this shortcoming, some authors have seen the advantages of this classification for smaller hernia types [41].

Some surgeons favour an additional radiography to verify the anatomical relationships of the HH within the chest [92,96]. Recently, some authors have reported their experience with high-resolution manometry in assessing the size of HHs [97]. These methods can be advantageous in some situations and can provide more insights. Unfortunately, in many health systems, the vast majority of patients will continue to be diagnosed only through the use of endoscopy, as the costs of a more detailed diagnostic work-up are often not covered by insurance companies [92].

Granderath et al. introduced a quantitative method to assess the width of the hiatus during laparoscopic surgery [98]. The “hiatal surface area” is measured and calculated intraoperatively, and some authors have found these parameters to be advantageous for deciding on the use of a mesh enforcement of the hiatal structures [98,99]. The possible disadvantage of this approach could be the fact that it cannot be used for preoperative planning, which could be valuable in order to involve the patient in informed consent.

The results of this study regarding the distribution of HH types show that there can be differences within the PEH subgroups. Data from publications where a more stringent taxonomy was applied showed that sliding Type I had an incidence of 80–90%, while Type II and Type IV were rather rare, with an incidence between 5 and 10% [23,25,27]. The most frequent type among the PEH subgroups was the mixed Type III. In the present review, these data were confirmed, with the majority of patients classified in sliding HH Type I. The distribution among the PEH subgroups confirmed the rarer incidence of Types II and IV, the true para-oesophageal types of HH. The mixed Type III occurred the most frequently among the PEHs associated with severe GERD, as shown in many studies [23,26,49,56,59,71,76]. A register series from Germany showed interesting results for 3042 HH patients [100]. The largest group of patients was the sliding Type I group (n = 2047); however, a different distribution among the PEH subgroups was noted. Among 996 patients with Types I, III, and IV, Type I accounted for 8.6%, Type III for only 28.0%, and Type IV for 45.6%, which was very high compared with other reports in the literature [1,2,3,4,5,6,7,8,9,10,11,12,13,14,15,16,17,18,19,20,21,22,23,24,25,26,27,28,29,30,31,32,33,34,35,36,37,38,39,40,41,42,43,44,45,46,47,48,49,50,51,52,53,54,55,56,57,58,59,60,61,62,63,64,65,66,67,68,69,70,71,72,73,74,75,76,77]. This discrepancy could be due to the fact that these data were collected in a registry in which surgeons could participate and send their data selectively.

This controversy underlines again the need to differentiate the different anatomical types of HH more accurately for the diagnosis and typing of HH. HH typing may allow for an assessment of the severity of the disease and thus provide the GERD patient with a prognosis. The precise type and size of HH may be important in determining the level of surgical experience necessary to perform the operation. Additionally, precise assessment and documentation may be valuable for further scientific evaluation and learning at any academic institution. The possible solution for improving the classification of HH must be based on endoscopy, which is performed during the diagnostic work-up in every single patient with an HH. Incorporating elements of artificial intelligence may support this. Recently, active learning (AL) systems have been established to help recognise and classify HH based on the endoscopic Hill classification during routine upper GI endoscopy, with remarkable results [101]. These systems could help to integrate endoscopic HH classifications without further time-consuming effort in daily endoscopic practice.

## 5. Conclusions

This review shows the increasing neglect of correct HH classification and taxonomy, with only a minority of publications using a stringent application. The 60 reports with detailed results confirmed the rare incidence of HH Types II and IV. Nevertheless, HH classification is important for the following reasons: first, it provides essential information regarding the pathophysiology and severity of the frequently associated GERD and subsequent therapeutic decision-making, including the prognosis of the GERD patient; second, the precise type and size of HH may be important in determining the level of surgical experience necessary to perform the operation; and, third, precise assessment and documentation may be valuable for further scientific evaluation at any academic institution.

## Figures and Tables

**Table 1:** Overview of different elements as determined in Akerlund’s, Allison’s, and Skinner’s publications regarding the classification of hiatal hernias.

Number	Definition of Element for Typing	Further Explanations for the Definition
1	True para-oesophageal hernia Akerlund 1926 [1]	The cardia remains at the level of the diaphragm. The hernia develops by rolling up through a small defect in the phreno-oesophageal membrane next to the oesophagus into the mediastinum.
2	Short oesophagus by congenital predisposition	This phenomenon was described in early experiences with HH and a short oesophagus; this element was only described in the 1930s and 1940s publications, and then it was dropped.
3	Short oesophagus acquired Collis	The association between oesophagitis and oesophageal shortening was recognised in the 1950s.
4	Sliding hernia in chest, which develops from small to larger hernias Akerlund 1926 [1]	The proximal stomach is sliding into the mediastinum; thus, the cardia is leaving the level of the diaphragm via the circumferential weakening of the phreno-oesophageal membrane.
5	Mixed type of hernia Akerlund 1926 [1]	This hernia develops from a sliding hernia with a limited size, in which the stomach migrates into the chest, with the complete proximal part of the stomach (cardia, fundus, and corpus) in the chest. With this process, the oesophagus can shorten.
6	Other intra-abdominal organs slide into the intrathoracic hernia Skinner and Belsey 1967 [23]	The development of the hernia is huge, causing, under favourable conditions, a migration of the colon, spleen, and/or pancreas into the chest.
7	Upside-down stomach Skinner and Belsey 1967 [23]	This is a further development of a true para-oesophageal hernia through a small defect in the phreno-oesophageal membrane, causing a further rolling up of the stomach in the chest. Since the cardia remains attached to the diaphragm, the stomach rotates around this fixation upside-down in the mediastinum.

**Table 2:** Overview of the distribution of patients within the groups and the corresponding publications.

Method of Data Demonstration	n Patients	Number of Publications	Publication in References
All types	8904	27	[10,13,15,17,18,21,33,36,38,41,55,62,64,70,74,75]
Selective PEH	3829	33	[34,35,37,39,40,42,43,44,45,46,47,48,49,50,51,52,53,54,56,57,58,59,60,61,62,63,65,66,67,68,69,71,72,73]
Total	12,733	60	[10,13,15,17,18,20,21,22,23,24,25,26,27,28,29,30,31,32,33,34,35,36,37,38,39,40,41,42,43,44,45,46,47,48,49,50,51,52,53,54,55,56,57,58,59,60,61,62,63,64,65,66,67,68,69,70,71,72,73,74,75]

**Table 3:** Distribution of patients with HH classified using Skinner and Belsey typing based on a review of 60 publications in the literature.

Application of Typing	Type I	Type II	Type III	Type IV
Typing of **complete HH cohort**: 8904 patients	7441	413	965	85
Distribution percentage	83.6%	4.3%	10.8%	0.9%
Typing of **selective PEH cohort**: 3829 patients	-	348	2951	530
Distribution percentage	-	9.1%	77.1%	13.8%
Comparison percentage of PEH among the complete HH cohort/n = 1463) (exclusion Type I)	-	413 28.3%	965 65.9%	85 5.8%

## Data Availability

The original contributions presented in the study are included in the article, further inquiries can be directed to the corresponding author.

## References

[1] Akerlund A.I. (1926). Hernia diaphragmatica hiatus oesophagei vom anatomischen und rontgenologischen gesichtspunkt. Acta Radiol..

[2] Guthrie D., Jones F.H. (1940). The frequency and diagnosis of hiatal hernia. Ann. Surg..

[3] Harrington S.W. (1945). The Surgical Treatment of the More Common Type of Diaphragmatic Hernia: Report of 404 Cases. Ann. Surg..

[4] Stapleton J.G. (1947). Oesophageal hiatus hernia. Can. Med. Assoc. J..

[5] Trueman K.R. (1947). Diagnosis and treatment of para-oesophageal hiatus hernia. Can. Med. Assoc. J..

[6] Allison P.R. (1948). Peptic ulcer of the oesophagus. Thorax.

[7] Olsen A.M., Harrington S.W. (1948). Esophageal hiatal hernias of the short esophagus type; etiologic and therapeutic considerations. J. Thorac. Surg..

[8] Merendino K.A., Varco R.L., Wangensteen O.H. (1949). Displacement of the Esophagus into a New Diaphragmatic Orifice in the Repair of Paraesophageal and Esophageal Hiatus Hernia. Ann. Surg..

[9] Stephens H.B. (1949). The problem of the acquired short esophagus; report of 18 patients. Calif. Med..

[10] Kaplan S. (1951). Oesophageal hiatus hernia. A clinical study of 45 cases. Postgrad. Med. J..

[11] Sweet R.H. (1952). Esophageal hiatus hernia of the diaphragm; the anatomical characteristics, technic of repair, and results of treatment in 111 consecutive cases. Ann. Surg..

[12] Barrett N.R. (1952). Hiatus hernia. Proc. R. Soc. Med..

[13] Macdougall J.T., Abbott A.C. (1957). Sliding hiatus hernia; a review of cases. Can. Med. Assoc. J..

[14] Barrett N.R. (1960). Hiatus hernia. Br. Med. J..

[15] Collis J.L. (1961). A review of surgical results in hiatus hernia. Thorax.

[16] Paulson D.L.M., Shaw R.R.M., Kee J.L.M. (1962). Esophageal hiatal diaphragmatic hernia and its complications. Ann. Surg..

[17] Borgeskov S., Pedersen O.T., Frederiksen T. (1964). Oesophageal Hiatal Hernia: A Radiological Follow-up. Thorax.

[18] Edwards D.A.W., Phillips S.F., Rowlands E.N. (1964). Clinical and Radiological Results of Repair of Hiatus Hernia. Br. Med. J..

[19] Brindley G.V. (1965). Surgical Treatment of Complications of Esopliageal Hiatal Hernia. Ann. Surg..

[20] Clagett O.T. (1966). Present concepts regarding the surgical treatment of oesophageal hiatal hernia. Ann. R. Coll. Surg. Engl..

[21] Krupp S., Rossetti M. (1966). Surgical treatment of hiatal hernias by fundoplication and gastropexy (Nissen repair). Ann. Surg..

[22] Zellos S. (1966). Surgical treatment of hiatal hernia, with particular reference to the transthoracic subdiaphragmatic approach. Thorax.

[23] Skinner D.B., Belsey R.H. (1967). Surgical management of esophageal reflux and hiatus hernia. Long-term results with 1,030 patients. J. Thorac. Cardiovasc. Surg..

[24] Hill L.D. (1967). An effective operation for hiatal hernia: An eight year appraisal. Ann. Surg..

[25] Nissen R., Pfeifer K. (1967). Aktuelle Probleme in der Chirurgie: Zwerchfellhernien.

[26] Borrie J. (1967). Surgical treatment of hiatal hernia: A 10-year survey. Thorax.

[27] Hamelmann H. (1968). Hiatal hernias in adults. Langenbecks Arch. Chir..

[28] Allison P.R. (1973). Hiatus hernia: (a 20-year retrospective survey). Ann. Surg..

[29] Hoffman E., Sumner M.C. (1973). A clinical and radiological review of 204 hiatal hernia operations. Thorax.

[30] Mokka R.E., Laitinen S., Punto L., Kairaluoma M.I., Pokela R., Kärkölä P., Huttunen R., Larmi T.K. (1976). Hiatal hernia repair. Ann. Chir. Gynaecol..

[31] Jacobsson S.I. (1976). Hiatal hernia. Follow-up of a ten-year material. Acta Chir. Scand. Suppl..

[32] Maher J.W., Hollenbeck J.I., Woodward E.R. (1978). An analysis of recurrent esophagitis following posterior gastropexy. Ann. Surg..

[33] Rossetti M. (1982). Indication et tactique chirurgicales dans le reflux et la hernie hiatale [Surgical indications and tactics in reflux and hiatal hernia]. Rev. Med. Suisse Rom..

[34] Pearson F.G., Cooper J.D., Ilves R., Todd T.R., Jamieson W.R. (1983). Massive hiatal hernia with incarceration: A report of 53 cases. Ann. Thorac. Surg..

[35] Hallissey M.T., Ratliff D.A., Temple J.G. (1992). Paraoesophageal hiatus hernia: Surgery for all ages. Ann. R. Coll. Surg. Engl..

[36] Kuster G.G., Gilroy S. (1993). Laparoscopic technique for repair of paraesophageal hiatal hernias. J. Laparoendosc. Surg..

[37] Menguy R. (1994). Le traitement chirugical des hernies hiatales par roulement avec volvulus intrathoracique de la totalité de l’estomac [Surgical treatment of paraesophageal hiatal hernia with total intrathoracic volvulus of the stomach]. Chirurgie.

[38] Jamieson G.G., Watson D.I., Britten-Jones R., Mitchell P.C., Anvari M. (1994). Laparoscopic Nissen fundoplication. Ann. Surg..

[39] Oddsdottir M., Franco A.L., Laycock W.S., Waring J.P., Hunter J.G. (1995). Laparoscopic repair of paraesophageal hernia. New access, old technique. Surg. Endosc..

[40] Myers G.A., Harms B.A., Starling J.R. (1995). Management of paraesophageal hernia with a selective approach to antireflux surgery. Am. J. Surg..

[41] Hill L.D., Kozarek R.A., Kraemer S.J., Aye R.W., Mercer C.D., Low D.E., Pope C.E. (1996). The gastroesophageal flap valve: In vitro and in vivo observations. Gastrointest. Endosc..

[42] Perdikis G., Hinder R.A., Filipi C.J., Walenz T., McBride P.J., Smith S.L., Katada N., Klingler P.J. (1997). Laparoscopic paraesophageal hernia repair. Arch. Surg..

[43] Edye M.B., Canin-Endres J., Gattorno F., Salky B.A. (1998). Durability of laparoscopic repair of paraesophageal hernia. Ann. Surg..

[44] Altorki N.K., Yankelevitz D., Skinner D.B. (1998). Massive hiatal hernias: The anatomic basis of repair. J. Thorac. Cardiovasc. Surg..

[45] Maziak D.E., Todd T.R., Pearson F.G. (1998). Massive hiatus hernia: Evaluation and surgical management. J. Thorac. Cardiovasc. Surg..

[46] Wu J.S., Dunnegan D.L., Soper N.J. (1999). Clinical and radiologic assessment of laparoscopic paraesophageal hernia repair. Surg. Endosc..

[47] Swanstrom L.L., Jobe B.A., Kinzie L.R., Horvath K.D. (1999). Esophageal motility and outcomes following laparoscopic paraesophageal hernia repair and fundoplication. Am. J. Surg..

[48] Luketich J.D., Raja S., Fernando H.C., Campbell W., Christie N.A., Buenaventura P.O., Weigel T.L., Keenan R.J., Schauer P.R. (2000). Laparoscopic repair of giant paraesophageal hernia: 100 consecutive cases. Ann. Surg..

[49] Dahlberg P.S., Deschamps C., Miller D.L., Allen M.S., Nichols F.C., Pairolero P.C. (2001). Laparoscopic repair of large paraesophageal hiatal hernia. Ann. Thorac. Surg..

[50] Champion J.K., Rock D. (2003). Laparoscopic mesh cruroplasty for large paraesophageal hernias. Surg. Endosc..

[51] Leeder P.C., Smith G., Dehn T.C. (2003). Laparoscopic management of large paraesophageal hiatal hernia. Surg. Endosc..

[52] Patel H.J., Tan B.B., Yee J., Orringer M.B., Iannettoni M.D. (2004). A 25-year experience with open primary transthoracic repair of paraesophageal hiatal hernia. J. Thorac. Cardiovasc. Surg..

[53] Andujar J.J., Papasavas P.K., Birdas T., Robke J., Raftopoulos Y., Gagné D.J., Caushaj P.F., Landreneau R.J., Keenan R.J. (2004). Laparoscopic repair of large paraesophageal hernia is associated with a low incidence of recurrence and reoperation. Surg. Endosc..

[54] Targarona E.M., Novell J., Vela S., Cerdán G., Bendahan G., Torrubia S., Kobus C., Rebasa P., Balague C., Garriga J. (2004). Mid term analysis of safety and quality of life after the laparoscopic repair of paraesophageal hiatal hernia. Surg. Endosc..

[55] Zaninotto G., Portale G., Costantini M., Rizzetto C., Guirroli E., Ceolin M., Salvador R., Rampado S., Prandin O., Ruol A. (2007). Long-term results (6–10 years) of laparoscopic fundoplication. J. Gastrointest. Surg..

[56] Boushey R.P., Moloo H., Burpee S., Schlachta C.M., Poulin E.C., Haggar F., Trottier D.C., Mamazza J. (2008). Laparoscopic repair of paraesophageal hernias: A Canadian experience. Can. J. Surg..

[57] Luketich J.D., Nason K.S., Christie N.A., Pennathur A., Jobe B.A., Landreneau R.J., Schuchert M.J. (2010). Outcomes after a decade of laparoscopic giant paraesophageal hernia repair. J. Thorac. Cardiovasc. Surg..

[58] Mittal S.K., Bikhchandani J., Gurney O., Yano F., Lee T. (2011). Outcomes after repair of the intrathoracic stomach: Objective follow-up of up to 5 years. Surg. Endosc..

[59] Pallabazzer G., Santi S., Parise P., Solito B., Giusti P., Rossi M. (2011). Giant hiatal hernias: Direct hiatus closure has an acceptable recurrence rate. Updates Surg..

[60] Carrott P.W., Hong J., Kuppusamy M., Kirtland S., Koehler R.P., Low D.E. (2012). Repair of giant paraesophageal hernias routinely produces improvement in respiratory function. J. Thorac. Cardiovasc. Surg..

[61] Lugaresi M., Mattioli S., Aramini B., D’Ovidio F., Di Simone M.P., Perrone O. (2013). The frequency of true short oesophagus in type II-IV hiatal hernia. Eur. J. Cardiothorac. Surg..

[62] Bjelović M., Babic T., Gunjić D., Veselinović M., Spica B. (2014). Laparoscopic repair of hiatal hernias: Experience after 200 consecutive cases. Srp. Arh. Celok. Lek..

[63] Latzko M., Borao F., Squillaro A., Mansson J., Barker W., Baker T. (2014). Laparoscopic repair of paraesophageal hernias. JSLS J. Soc. Laparosc. Robot. Surg..

[64] Chang C.G., Thackeray L. (2016). Laparoscopic Hiatal Hernia Repair in 221 Patients: Outcomes and Experience. JSLS J. Soc. Laparosc. Robot. Surg..

[65] Stringham J.R., Phillips J.V., McMurry T.L., Lambert D.L., Jones D.R., Isbell J.M., Lau C.L., Kozower B.D. (2017). Prospective study of giant paraesophageal hernia repair with 1-year follow-up. J. Thorac. Cardiovasc. Surg..

[66] Mertens A.C., Tolboom R.C., Zavrtanik H., Draaisma W.A., Broeders I.A.M.J. (2019). Morbidity and mortality in complex robot-assisted hiatal hernia surgery: 7-year experience in a high-volume center. Surg. Endosc..

[67] Shea B., Boyan W., Decker J., Almagno V., Binenbaum S., Matharoo G., Squillaro A., Borao F. (2019). Emergent Repair of Paraesophageal Hernias and the Argument for Elective Repair. JSLS J. Soc. Laparosc. Robot. Surg..

[68] Dara V., Croo A., Peirsman A., Pattyn P. (2019). Necessity of fundoplication and mesh in the repair of the different types of paraesophageal hernia. Acta Gastroenterol. Belg..

[69] Sorial R.K., Ali M., Kaneva P., Fiore J.F., Vassiliou M., Fried G.M., Feldman L.S., Ferri L.E., Lee L., Mueller C.L. (2020). Modern era surgical outcomes of elective and emergency giant paraesophageal hernia repair at a high-volume referral center. Surg. Endosc..

[70] Fuchs K.H., Breithaupt W., Varga G., Babic B., Schulz T., Meining A. (2022). Primary laparoscopic fundoplication in selected patients with gastroesophageal reflux disease. Dis. Esophagus.

[71] Rodríguez-Luna M.R., Pizzicannella M., Fiorillo C., Almuttawa A., Lapergola A., Mutter D., Marrescaux J., Dallemagne B., Perretta S. (2022). Impact of surgical repair on type IV paraesophageal hernias (PEHs). Surg. Endosc..

[72] D’Elia M.A., Ahmadi N., Jarrar A., Neville A., Mamazza J. (2022). Paraesophageal hernia repair in elderly patients: Outcomes from a 10-year retrospective study. Can. J. Surg..

[73] Amundson J.R., Kuchta K., Wu H., VanDruff V.N., Haggerty S.P., Linn J., Ujiki M.B. (2023). A 13-year experience with biologic and biosynthetic absorbable mesh reinforced laparoscopic paraesophageal hernia repair. Surg. Endosc..

[74] Salvador R., Vittori A., Capovilla G., Riccio F., Nezi G., Forattini F., Provenzano L., Nicoletti L., Moletta L., Costantini A. (2023). Antireflux Surgery’s Lifespan: 20 Years After Laparoscopic Fundoplication. J. Gastrointest. Surg..

[75] Giulini L., Razia D., Latorre-Rodríguez A.R., Shacker M., Csucska M., Mittal S.K. (2023). Surgical Repair of Large Hiatal Hernias: Insight from a High-Volume Center. J. Gastrointest. Surg..

[76] Furnée E.J., Draaisma W.A., Gooszen H.G., Hazebroek E.J., Smout A.J., Broeders I.A. (2011). Tailored or routine addition of an antireflux fundoplication in laparoscopic large hiatal hernia repair: A comparative cohort study. World J. Surg..

[77] Dallemagne B., Kohnen L., Perretta S., Weerts J., Markiewicz S., Jehaes C. (2011). Laparoscopic repair of paraesophageal hernia. Long-term follow-up reveals good clinical outcome despite high radiological recurrence rate. Ann. Surg..

[78] Stylopoulos N., Rattner D.W. (2005). The history of hiatal hernia surgery: From Bowditch to laparoscopy. Ann. Surg..

[79] Gyawali C.P., Kahrilas P.J., Savarino E., Zerbib F., Mion F., Smout A.J.P.M., Vaezi M., Sifrim D., Fox M.R., Vela M.F. (2018). Modern diagnosis of GERD: The Lyon Consensus. Gut.

[80] Kahrilas P.J., Kim H.C., Pandolfino J.E. (2008). Approaches to the diagnosis and grading of hiatal hernia. Best. Pract. Res. Clin. Gastroenterol..

[81] DeMeester T.R., Yeo C.J., DeMeester S.R., Fadden D.W.M. (2019). Etiology and Natural History of Gastroesophageal Reflux Disease and Predictors of progressive Disease. Shackelford’s Surgery of the Alimentary Tract.

[82] Fuchs K.H., DeMeester T.R., Otte F., Broderick R.C., Breithaupt W., Varga G., Musial F. (2021). Severity of GERD and disease progression. Dis. Esophagus.

[83] DeMeester T.R., Johnson L.F., Joseph G.J., Toscano M.S., Hall A.W., Skinner D.B. (1976). Patterns of gastroesophageal reflux in health and disease. Ann. Surg..

[84] Costantini M., Crookes P.F., Bremner R.M., Hoeft S.F., Ehsan A., Peters J.H., Bremner C.G., DeMeester T.R. (1993). Value of physiologic assessment of foregut symptoms in a surgical practice. Surgery.

[85] Tack J., Caenepeel P., Arts J., Lee K.J., Sifrim D., Janssens J. (2005). Prevalence of acid reflux functional dyspepsia and its association with symptom profile. Gut.

[86] Rydberg L., Ruth M., Abrahamsson H., Lundell L. (1999). Tailoring antireflux surgery: A randomized clinical trial. World J. Surg..

[87] Fibbe C., Layer P., Keller J., Strate U., Emmermann A., Zornig C. (2001). Esophageal motility in reflux disease before and after fundoplication: A prospective, randomized, clinical, and manometric study. Gastroenterology.

[88] Dallemagne B., Weertz J., Markiewicz S., Dewandre J.M., Wahlen C., Monami B., Jehaes C. (2006). Clinical results of laparoscopic fundoplication ten years after surgery. Surg. Endosc..

[89] Frantzides C.T., Madan A.K., Carlson M.A., Stavropoulos G.P. (2002). A prospective, randomized trial of laparoscopic polytetrafluoroethylene (PTFE) patch repair vs simple cruroplasty for large hiatal hernia. Arch. Surg..

[90] Granderath F.A., Schweiger U.M., Kamolz T., Pasiut M., Haas C.F., Pointner R. (2002). Laparoscopic antireflux surgery with routine mesh-hiatoplasty in the treatment of gastroesophageal reflux disease. J. Gastrointest. Surg..

[91] Bonavina L., DeMeester T., Fockens P., Dunn D., Saino G., Bona D., Lipham J., Bemelman W., Ganz R.A. (2010). Laparoscopic sphincter augmentation device eliminates reflux symptoms and normalizes esophageal acid exposure: One- and 2-year results of a feasibility trial. Ann. Surg..

[92] Jobe B.A., Richter J.E., Hoppo T., Peters J.H., Bell R., Dengler W.C., DeVault K., Fass R., Gyawali C.P., Kahrilas P.J. (2013). Preoperative diagnostic workup before antireflux surgery: An evidence and experience-based consensus of the Esophageal Diagnostic Advisory Panel. J. Am. Coll. Surg..

[93] Linke G.R., Gehrig T., Hogg L.V., Göhl A., Kenngott H., Schäfer F., Fischer L., Gutt C.N., Müller-Stich B.P. (2014). Laparoscopic mesh-augmented hiatoplasty without fundoplication as a method to treat large hiatal hernias. Surg. Today.

[94] DeMeester S.R. (2013). Laparoscopic paraesophageal hernia repair: Critical steps and adjunct techniques to minimize recurrence. Surg. Laparosc. Endosc. Percutan Tech..

[95] Gerdes S., Schoppmann S.F., Bonavina L., Boyle N., Müller-Stich B.P., Gutschow C.A., Hiatus Hernia Delphi Collaborative Group (2023). Management of paraesophageal hiatus hernia: Recommendations following a European expert Delphi consensus. Surg. Endosc..

[96] Fuchs K.H., Babic B., Breithaupt W., Dallemagne B., Fingerhut A., Furnee E., Granderath F., Horvath O.P., Kardos P., Pointner R. (2014). EAES recommendations for the management of Gastroesophageal reflux Disease. Surg. Endosc..

[97] Tolone S., Savarino E., Zaninotto G., Gyawali C.P., Frazzoni M., de Bortoli N., Frazzoni L., Del Genio G., Bodini G., Furnari M. (2018). High-resolution manometry is superior to endoscopy and radiology in assessing and grading sliding hiatal hernia: A comparison with surgical in vivo evaluation. United Eur. Gastroenterol. J..

[98] Granderath F.A., Schweiger U.M., Pointner R. (2007). Laparoscopic antireflux surgery: Tailoring the hiatal closure to the size of hiatal surface area. Surg. Endosc..

[99] Grubnik V.V., Malynovskyy A.V. (2013). Laparoscopic repair of hiatal hernias: New classification supported by long-term results. Surg. Endosc..

[100] Köckerling F., Zarras K., Adolf D., Kraft B., Jacob D., Weyhe D., Schug-Pass C. (2020). What Is the Reality of Hiatal Hernia Management? A Registry Analysis. Front. Surg..

[101] Kafetzis I., Fuchs K.H., Sodmann P., Troya J., Zoller W., Meining A., Hann A. (2024). Efficient artificial intelligence-based assessment of the gastroesophageal valve with Hill classification through active learning. Sci. Rep..

